# Cytomegalovirus Infection Is Associated with Increased Disease Activity and Worse Long-Term Course in Ulcerative Colitis

**DOI:** 10.7150/ijms.139439

**Published:** 2026-09-03

**Authors:** Richard Vollenberg, Benedikt Blömer, Clara Zippel, Markus Strauss, Eva Lorentzen, Jonel Trebicka, Phil-Robin Tepasse

**Affiliations:** 1 Department of Internal Medicine B for Gastroenterology, Hepatology, Endocrinology and Clinical Infectiology, Germany.; 2 Department of Cardiology I-Coronary and Peripheral Vascular Disease, Heart Failure Medicine, University Hospital Muenster, 48149 Münster, Germany.; 3 Department of Cardiology, Faculty of Health, School of Medicine, University Witten/Herdecke, 58448 Witten, Germany.; 4 Institute of Virology, University of Muenster, Domagkstr. 40, D-48149 Muenster, Germany.

**Keywords:** ulcerative colitis, cytomegalovirus, CMV infection, CMV reactivation, tissue PCR, endoscopic Mayo score, colectomy, advanced therapies, treatment failure, inflammatory bowel disease

## Abstract

**Background:**

Human cytomegalovirus (CMV) infection is frequently detected in patients with ulcerative colitis (UC), particularly in severe or refractory disease. Its impact on disease activity, long-term course, and clinical outcomes remains controversial.

**Methods:**

In this retrospective single-center cohort study conducted at the University Hospital Münster (Germany), adult UC patients undergoing colonoscopy between May 2002 and May 2024 were included. CMV status was determined by tissue-based PCR from colonic biopsies at baseline colonoscopy (BL). Patients with at least one follow-up colonoscopy (FU) were analyzed. Clinical and endoscopic disease activity (total Mayo score [tMayo], endoscopic Mayo score [eMayo]), remission rates, treatment exposure, and outcomes were compared between patients with CMV-positive and CMV-negative biopsies at BL and FU.

**Results:**

A total of 181 UC patients were included (63 CMV-positive, 118 CMV-negative biopsies). At BL, CMV-positive patients had significantly higher endoscopic disease activity (eMayo, *p* = .03) and numerically higher clinical activity. At FU (median interval 34 vs. 45 months), both groups showed improvement; however, CMV-positive patients continued to exhibit significantly higher disease activity (tMayo *p* = .001; eMayo *p* < .001). Rates of clinical/endoscopic remission were significantly lower in CMV-positive patients at both BL and FU. No significant association was observed between CMV status of biopsies and colectomy rates (*p* = .21). Antiviral therapy was administered in 60% of CMV-positive patients and was associated with higher baseline disease activity but not with differences at FU.

**Conclusions:**

Molecular detection of CMV in the intestinal mucosa is associated with increased mucosal inflammation and a less favorable long-term disease course in UC, but has no effect on hard outcome parameters such as colectomy. Antiviral therapy has no effect on clinical outcome parameters. These findings indicate that further studies are needed to clarify the pathophysiological influences of CMV infection on disease course in ulcerative colitis and its impact on the efficacy of advanced therapies.

## 1. Introduction

Human cytomegalovirus (CMV, Human Herpesvirus 5) belongs to the herpesvirus family, establishes latent, persistent infections following primary infection, and is ubiquitously distributed 1. The seroprevalence rates of CMV infection exhibit a wide range, varying from 40 % in adults living in highly developed countries to more than 95 % in populations with a low socioeconomic status 2. Furthermore, these rates have been found to increase with age. While periodical reactivation of latent CMV rarely leads to clinically apparent disease in immunocompetent individuals, immunosuppressed patients carry a substantial risk of manifest CMV disease linked to inflammatory processes following reactivation 3-5. Ulcerative colitis (UC) is a chronic, relapsing inflammatory bowel disease characterized by continuous mucosal inflammation of the colon and often requires long-term immunosuppressive and biologic therapy 6. The first description of CMV colitis in UC patients was reported in 1961 by Powell et al. 3. Epidemiological studies indicate that CMV reactivation occurs in a substantial proportion of hospitalized UC patients with severe flares, particularly in those receiving high-dose corticosteroids or other immunosuppressive agents 7,8.

However, the impact of CMV infection or reactivation on the disease course of ulcerative colitis, as well as its role in triggering or aggravating disease flares and contributing to therapy resistance, remains a matter of ongoing debate. Furthermore, its influence on the length of hospital stay, the need for colectomy, and mortality rates is still unclear 9-11. It is also controversial whether CMV infection is causally involved in the severity of inflammation or rather represents an incidental finding or marker of the underlying disease severity 6,12,13. Current guidelines therefore recommend targeted CMV diagnostics—preferably tissue-based assays—in patients with steroid-refractory or steroid-dependent UC; however, thresholds for antiviral treatment and the prognostic significance of low-level CMV DNA in the affected organ remain uncertain 6,8,14,15. Thus, CMV infection continues to pose significant challenges for attending gastroenterologists.

Against this background, our retrospective cohort study aimed to investigate the relationship between the PCR-confirmed presence of CMV DNA in the colon and disease severity, clinical and endoscopic course, as well as the need for therapy escalation as an indicator of treatment failure in a tertiary care UC population.

## 2. Materials and Methods

### 2.1. Study design

This retrospective single-center study was conducted at the University Hospital Münster (Germany) and included adult patients treated either as inpatients or outpatients. The observation period ranged from May 2002 to May 2024.

In the CMV-positive cohort, patients were included if they had a CMV-positive biopsy (CMV DNA detection above the cut-off by real-time polymerase chain reaction, RT-PCR) during the first colonoscopy performed at our center (n = 285). After excluding all patients without ulcerative colitis or with incomplete documentation, 72 patients remained who had undergone at least two endoscopic procedures (colonoscopies or sigmoidoscopies) at our center. Among these, 9 patients underwent colectomy during the observation period (last endoscopy: sigmoidoscopy after colectomy). Ultimately, 63 UC patients had a documented initial colonoscopy with CMV-positive biopsy (baseline, BL) and at least one additional complete colonoscopy (last colonoscopy performed at our center, follow-up colonoscopy, FU).

Additionally, patients with a CMV-negative biopsy during the baseline endoscopy (n = 265) were identified within the observation period. After excluding patients without UC, with incomplete documentation, without at least two endoscopic procedures (colonoscopies or sigmoidoscopies), or with documented CMV infection/reinfection in medical reports or follow-up endoscopies, 127 patients remained. Among these, 9 patients also underwent colectomy during the observation period. Considering only patients with at least two complete colonoscopies (BL and FU), the final CMV-negative cohort comprised 118 patients (Supplemental Figure S1).

### 2.2. Definitions and variables

CMV status was determined using colonic biopsies obtained from endoscopically suspicious areas for CMV infection/reactivation. Biopsies were subjected to a digestion process that involved the addition of 80 µl of proteinase K solution and 720 µl of ATL buffer (Qiagen, Hilden, Germany). The samples were then incubated at 56°C for a period of at least four hours, until complete dissolution of the tissue. Viral DNA was extracted from the solutions and qualitative real-time PCR (RT-PCR) was performed subsequently on the eluates using CMV-specific primers and probes according to the instructions of the test kits' manufacturers. As stipulated, negative, internal extraction, and positive controls were incorporated into RT-PCR runs. Concurrently, established CMV standards were amplified to verify the detection of CMV DNA within a linear range. CMV viral load (copies/ml eluate) was descriptively categorized as low (<1,000 copies/ml), medium (1,000-10,000 copies/ml), and high (>10,000 copies/ml). It is important to note that the absolute numerical values have limited methodological validity due to uncontrollable dilution effects and biopsies constituting non-standardised diagnostic materials. Consequently, they are used for descriptive purposes only, in order to illustrate the distribution of copies/ml. Furthermore, PCR test kits were obtained from different manufacturers over the 22-year observation period, demanding caution when directly comparing quantitative results.

Patients who were considered to have a clinically relevant colonic CMV detection based on the overall assessment of findings (endoscopic findings, clinical disease severity and current medical therapy) were treated with either valganciclovir (oral), ganciclovir (intravenous), or a sequential therapy (initial inpatient treatment with intravenous ganciclovir followed by oral valganciclovir) 7,8. The duration and dose of treatment was not standardized across patients.

At the time points of BL and FU colonoscopy, in addition to general characteristics, the total Mayo score (tMayo, 0-12 points), the endoscopic Mayo score (eMayo, 0-3 points), and therapy burden were assessed 16. Endoscopic remission (eRemission) was defined as an eMayo score of 0, and clinical/endoscopic remission was defined as a tMayo score of 0-2 points. All data were collected from electronic medical records and endoscopy databases and analyzed in pseudonymized form in accordance with local data protection regulations (Figure 1).

### 2.3. Statistical analysis

Continuous variables are presented as median with interquartile range (IQR) for non-normally distributed data. Categorical variables are summarized as absolute numbers and percentages. Between-group comparisons (e.g., patients with CMV-positive vs. CMV-negative biopsies) were performed using the independent samples t-test for continuous variables with approximate normal distribution and homogeneity of variances, and the Mann-Whitney U test for non-normally distributed continuous variables. Normality of continuous variables was assessed using the Shapiro-Wilk test together with visual inspection of histograms and Q-Q plots; none of the continuous variables analyzed in this cohort met the criteria for a normal distribution, and the t-test was therefore not applied to any variable. All between-group comparisons for continuous variables reported in this study were accordingly performed using the Mann-Whitney U test. Categorical variables were compared using the chi-squared test when expected cell counts were adequate; otherwise, Fisher's exact test was applied. For within-patient comparisons between BL and FU measurements, the paired t-test, Mann-Whitney U test, or Wilcoxon signed-rank test was used for continuous variables, depending on the distribution of the paired differences. As the paired differences for all continuous variables were likewise non-normally distributed, the Wilcoxon signed-rank test was used for all within-patient comparisons of continuous variables, and the paired t-test was not applied to any variable. For paired samples with dichotomous variables, McNemar's test was used. The association between ordinally scaled variables was assessed using Spearman's rank correlation coefficient. All statistical tests were two-sided, and a *p* value < 0.05 was considered statistically significant. Statistical analyses were performed using IBM SPSS Statistics, Version 31 (IBM Corp., Armonk, NY, USA).

The study protocol was reviewed and approved by the local ethics committee of the Medical Faculty of the University of Münster (reference number 2020-566-f-S and amendment dated 04 June 2024). Given the retrospective design and use of pseudonymized data, the requirement for individual informed consent was waived in accordance with institutional and national regulations.

## 3. Results

### 3.1 Study population

A total of 181 UC patients were included, of whom 63 were CMV-positive and 118 CMV-negative at BL colonoscopy (defined as a positive CMV PCR result from colonic biopsies, irrespective of serological CMV status). During the study period, colectomy occurred in 9 patients per group (CMV-positive 12.5% vs. CMV-negative 7.1%; *p* = .21).

CMV-positive UC patients were older than CMV-negative patients at study inclusion (median 41 years [IQR 29-55] vs. 35 years [IQR 25-47]; *p* = .006, Table 1). The proportion of male patients was similar between groups (CMV-negative 59% vs. CMV-positive 60%; *p* = .896). The distribution according to the Montreal classification showed no significant differences (*p* = .185). Disease duration at BL colonoscopy did not differ significantly between patients with CMV-positive (median 30 months [IQR 9-85]) and CMV-negative biopsies (median 40 months [IQR 12-113]; *p* = .51). UC treatment at baseline was comparable between groups, with similar use of oral 5-ASA, rectal 5-ASA, oral prednisolone, rectal prednisolone, azathioprine, and anti-TNF agents (all *p* > .05). The use of other advanced therapies, including combination therapy with azathioprine and anti-TNF agents, vedolizumab, ustekinumab, ozanimod, and JAK inhibitors, was low and did not differ significantly between groups.

Most patients were biologic-naïve at the time of BL colonoscopy (CMV-negative 88% vs. CMV-positive 79%; *p* = .123). CMV-positive patients had significantly higher eMayo (*p* = .03) and tMayo scores (*p* = .011) compared with CMV-negative UC patients. Among CMV-positive patients, 40% had low CMV copies (<1,000 copies/ml), 17% had medium copies (1,000-10,000 copies/ml), and 43% had high CMV copies (>10,000 copies/ml). Of these CMV-positive patients (n = 63), 60% received antiviral therapy (2% ganciclovir, 19% valganciclovir, 40% sequential therapy with ganciclovir followed by valganciclovir). The median cumulative duration of antiviral treatment was 32 days (IQR 28-56). The indication to initiate CMV therapy was made in line with current guideline recommendations as an individualized case-by-case decision based on the presence of a relevant CMV finding, in the absence of clearly defined CMV cut-off values.

The median interval between BL and FU colonoscopy was 34 months (IQR 7-93) in CMV-positive patients versus 45 months (IQR 16-107) in CMV-negative patients (*p* = .114). Compared with BL colonoscopy, significantly fewer CMV-negative patients were receiving systemic prednisolone therapy at the time of FU colonoscopy (*p* = .006), whereas no significant difference was observed in CMV-positive patients (*p* = .406). In addition, at the time of FU colonoscopy, patients had significantly more previous failures of advanced therapy compared with BL colonoscopy (*p* < .001).

At FU colonoscopy, there were no significant differences between CMV-positive and CMV-negative UC patients regarding current immunosuppressive therapy or the number of previous failures of advanced therapy (all *p* > .05, Table 2). The eMayo score was numerically higher in CMV-positive patients, although not statistically significant (*p* = .086), whereas the tMayo score was significantly higher in CMV-positive compared with CMV-negative patients (*p* = .001).

### 3.2 Clinical and endoscopical outcomes by CMV Status

UC patients with CMV-DNA positive biopsies had a numerically higher, but not statistically significant, tMayo score at BL colonoscopy compared with patients with CMV-negative biopsies (median 8 [IQR 6-10] vs. 7 [IQR 4-10]; p = .051). At FU colonoscopy, CMV-positive patients had a significantly higher tMayo score (median 6 [IQR 1-10] vs. 2 [IQR 0-6]; p = .001). Both CMV-negative and CMV-positive UC patients showed significant improvement in tMayo scores at FU colonoscopy (CMV-negative: BL median 7 [IQR 4-10] vs. FU median 2 [IQR 0-6], p < .001; CMV-positive: BL median 8 [IQR 6-10] vs. FU median 6 [IQR 1-10], p < .001) (Figure 2a).

CMV-positive patients had significantly higher eMayo scores compared with CMV-negative UC patients at both BL colonoscopy (median 2 [IQR 2-3] vs. 2 [IQR 1-3]; p = .03) and FU colonoscopy (median 2 [IQR 0-3] vs. 1 [IQR 1-2]; p < .001). Both CMV-positive and CMV-negative UC patients showed significant improvement in eMayo scores at FU colonoscopy (CMV-positive: BL median 2 [IQR 2-3] vs. FU median 2 [IQR 0-3], p < .001; CMV-negative: BL median 2 [IQR 1-3] vs. FU median 1 [IQR 1-2], p < .001) (Figure 2b).

Regarding endoscopic remission (eRemission, defined as eMayo score of 0), significantly more CMV-negative patients were in remission at BL colonoscopy (CMV-negative 14% vs. CMV-positive 5%; p = .036). At FU colonoscopy, there was no significant difference in eRemission between groups (CMV-negative 40% vs. CMV-positive 27%; p = .082). However, both CMV-positive and CMV-negative UC patients showed significant improvement in eRemission rates at FU colonoscopy (CMV-negative: BL 14% vs. FU 40%, p < .001; CMV-positive: BL 5% vs. FU 27%, p < .001) (Figure 2c).

In combined clinical and endoscopic remission (ceRemission, defined as tMayo score 0-2), significantly more CMV-negative UC patients were in remission at both BL and FU colonoscopy (BL: 15% vs. 8%, p < .05; FU: 56% vs. 32%, p < .001). Both groups showed significant improvement in ceRemission rates at FU colonoscopy (CMV-negative: BL 15% vs. FU 56%, p < .001; CMV-positive: BL 8% vs. FU 32%, p < .001) (Figure 2c).

### 3.3. Antiviral therapy in CMV-positive UC patients

CMV-positive patients who received antiviral therapy (38/63) had significantly higher eMayo (median 3 [IQR 2-3] vs. 2 [IQR 2-3], p = .032) and tMayo scores (median 9 [IQR 7-11] vs. 7 [IQR 5-9], p = .01) at the time of BL colonoscopy compared with those who did not receive antiviral therapy. At FU colonoscopy, no significant differences were observed between the two groups with regard to eMayo (p = .534) or tMayo (p = .765).

There was no significant correlation between CMV copies detected in biopsies and eMayo or tMayo scores at either BL or FU colonoscopy in CMV-positive UC patients (BL: eMayo p = .348, tMayo p = .419; FU: eMayo p = .697, tMayo p = .763).

## 4. Discussion

In this monocentric, retrospective cohort study, we analyzed the clinical and endoscopic disease course of ulcerative colitis depending on CMV status of biopsies at the time of the first colonoscopy performed at our center.

We were able to demonstrate that CMV-positive UC patients (positive CMV PCR from colonic biopsies) showed significantly greater inflammation at initial colonoscopy compared with CMV-negative patients (negative CMV PCR from colonic biopsies; CMV serology was not available for all patients, but only those with negative serology and no evidence of acute CMV infection or reinfection were included, eMayo *p* = .03). Compared with BL colonoscopy, both eMayo and tMayo scores improved at FU colonoscopy (the last endoscopy performed at our center) in both groups; however, CMV-positive patients continued to exhibit significantly higher eMayo and tMayo scores at FU (*p* < .001 and *p* = .001, respectively). These findings suggest a significant association between the detection of CMV in biopsies obtained from UC patients and more pronounced mucosal inflammation as well as a less favorable long-term disease course.

Regarding the epidemiology of CMV infection or reactivation in UC patients, patients with CMV-positive biopsies in our cohort were significantly older than those without detectable CMV DNA levels, in line with previous studies (*p* = .006) **6,17**. While earlier studies have suggested an association between female sex and pancolitis with an increased risk of CMV reactivation in UC patients, we were unable to confirm this in our cohort (sex *p* = .896; disease extent according to Montreal classification *p* = .185) **6,18,19**, possibly due to the limited sample size.

The impact of CMV infection or reactivation on exacerbation of UC flares remains unclear. While some studies did not demonstrate a correlation between the endoscopic Mayo score and CMV DNA detection in the peripheral blood or colonic biopsies, others reported higher rates of severe endoscopic disease in patients with CMV antigenemia **6,20-22**. Two more recent studies defining CMV colitis by the presence of inclusion bodies or CMV DNA in colonic biopsies demonstrated higher disease activity in CMV-positive patients, reflected by both total Mayo score and endoscopic Mayo subscore **6,23,24**. These findings are consistent with our results, which are also based on CMV DNA detection in colonic biopsies.

The influence of CMV infection on relapse, natural disease course, prognosis, and response to advanced therapies remains controversial. In our study, CMV-positive patients continued to show significantly higher endoscopic (eMayo) and clinical activity (tMayo score) after a median follow-up of 34 months compared with CMV-negative patients. This supports findings by Xiao et al., who demonstrated a significantly increased risk of UC relapse in CMV-positive patients over a 24-month observation period **25,26**.

There is stronger evidence for an association between CMV infection and steroid resistance in acute severe UC **13,24,27**. However, it remains unclear whether steroid resistance is directly caused by CMV infection or reactivation, or represents a surrogate marker of severe inflammation **6**. In our study, no significant differences in the prevalence of prednisolone therapy were observed depending on the CMV status of colonic biopsies, possibly due to the limited sample size.

The impact of CMV infection on hard endpoints such as colectomy remains controversial **28,29**. While some retrospective case series **30,31** and multicenter cohort studies suggest that CMV reactivation in severe UC may be associated with an increased short-term risk of colectomy **6,23,32**, other studies have not demonstrated a significant association **14,33,34**. In our cohort, no significant difference in colectomy rates was observed according to baseline CMV status (*p* = .21) during the study period (between BL and FU colonoscopy).

In 38 of 63 patients with positive CMV detection at BL, antiviral therapy was administered. The decision to initiate antiviral therapy was made by the treating physician based on an overall assessment of all clinical findings and is in line with current German S3 guidelines for ulcerative colitis **7**. In our cohort, CMV-positive patients who received antiviral therapy had significantly higher disease activity at BL endoscopy compared with those without antiviral treatment. At FU colonoscopy, no significant differences in disease severity were observed between CMV-positive UC patients with and without antiviral therapy. Thus, we were unable to demonstrate a clear effect of antiviral treatment on clinical outcome.

In particular, CMV infection or reactivation should be excluded in therapy-refractory or steroid-refractory disease courses and treated if confirmed. However, there is still no diagnostic gold standard for CMV colitis **7**. Tissue-based PCR has a higher sensitivity compared with immunohistochemistry and blood-based diagnostic methods **35**, but may also detect low-level viral replication without clinical relevance, which does not benefit from antiviral therapy **29**. Furthermore, tissue CMV PCR may yield false-negative results, particularly in the setting of inadequate or non-representative biopsies **36**. In order to verify virus-induced disease processes inside the organ, it appears essential to assess CMV DNA levels concomitantly in the peripheral blood and in biopsies. As a quantitative CMV threshold for initiating antiviral therapy has not been established yet, treatment decisions remain individualized, taking into account disease course and clinical context **7,28,29**.

A limitation of this study is its retrospective single-center design. Furthermore, CMV biopsies were not systematically reassessed at FU colonoscopy, as the focus was on evaluating disease course based on CMV status at BL colonoscopy. Moreover, the CMV serostatus had not been systematically analyzed, which precluded the comparison of seropositive and seronegative patients without signs of virus reactivation in the affected organ. In addition, CMV-positive patients were significantly older than CMV-negative patients at study inclusion. As age may independently influence disease severity, treatment response, and long-term outcomes, this baseline imbalance represents a potential confounder that should be taken into account when interpreting the observed differences between groups.

## 5. Conclusions

In summary, this study establishes tissue PCR-confirmed CMV infection or reactivation within the colon as an important marker of severe mucosal inflammation in ulcerative colitis and demonstrates that these patients exhibit significantly higher eMayo and tMayo scores even after a median follow-up of 34 months. These findings complement current guideline concepts by supporting targeted CMV diagnostics in severe or refractory UC, a selective use of antiviral therapy, and the continued central role of clinical and endoscopic remission as primary treatment goals in UC management.

## Supplementary Material

Supplementary figure.

## Figures and Tables

**Figure 1:**
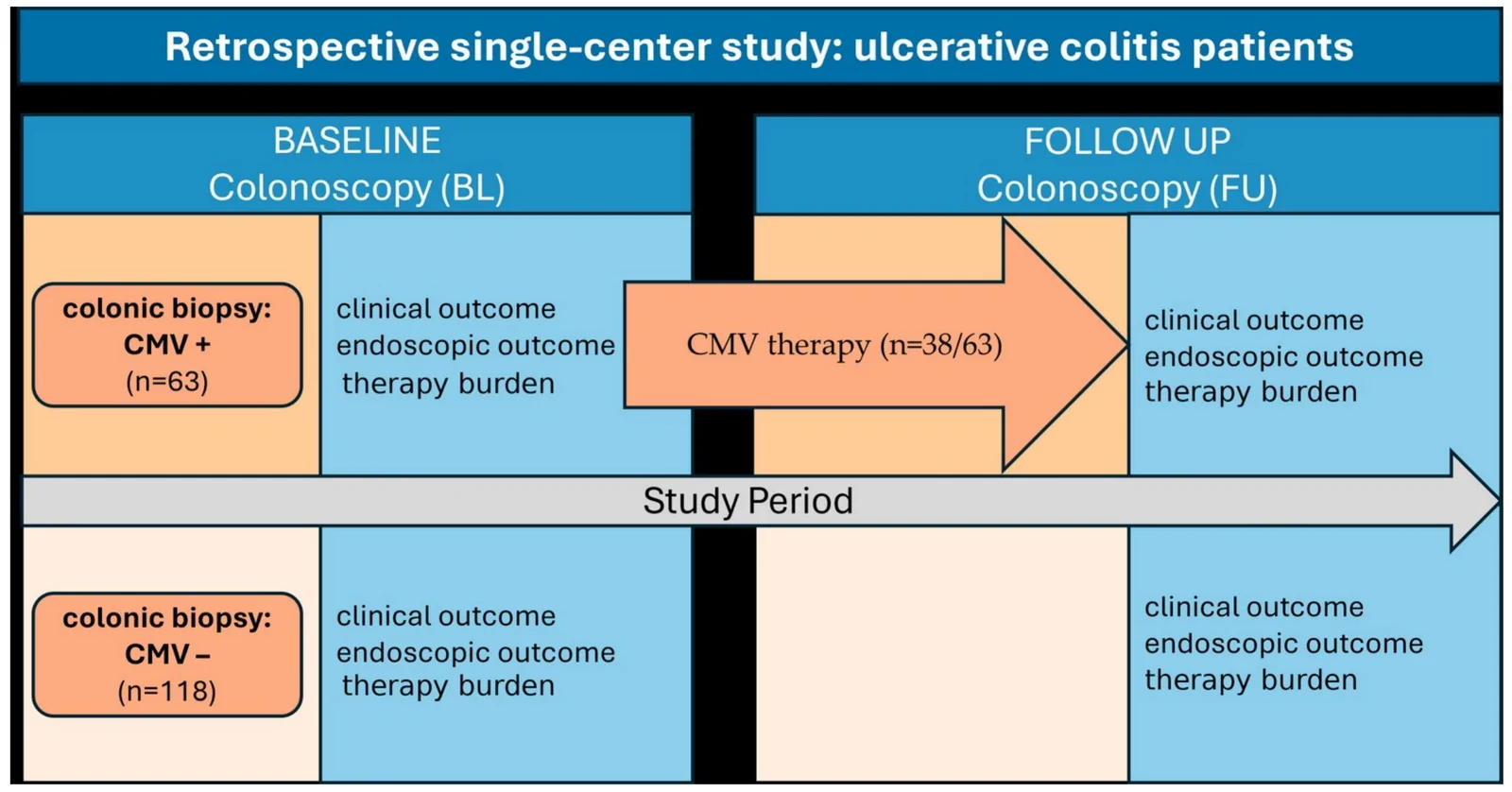
Study flow chart. CMV therapy was administered with ganciclovir and/or valganciclovir. Baseline colonoscopy (BL), follow-up colonoscopy (FU); CMV, cytomegalovirus; +, positive; -, negative; n, number.

**Figure 2:**
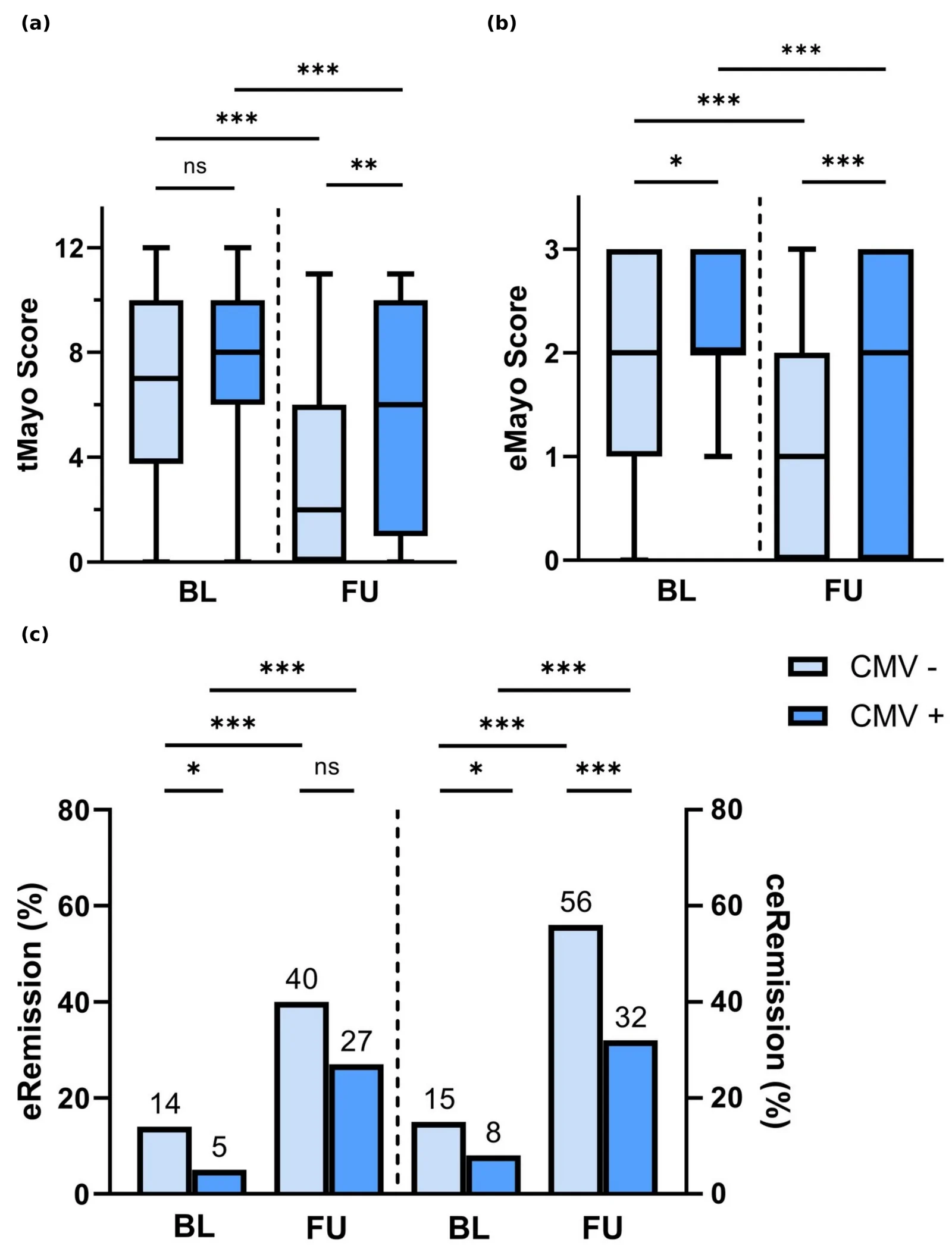
Clinical and endoscopic disease course of UC patients according to CMV status of colonic biopsies presented as Tukey boxplots. Total Mayo score (tMayo, range 0-12 points) in CMV-negative and CMV-positive UC patients at the time of BL and FU colonoscopy (a); endoscopic Mayo score (eMayo, range 0-3) according to CMV status at BL and FU colonoscopy (b); and percentage (%) of endoscopic and clinical/endoscopic remission (eRemission, ceRemission) according to CMV status at BL and FU colonoscopy (c). Endoscopic remission was defined as an eMayo score of 0, and clinical/endoscopic remission was defined as a tMayo score of 0-2 points. p values for unpaired continuous data were calculated using the Mann-Whitney U test, and paired continuous data were analyzed using the Wilcoxon signed-rank test. Nominal variables were analyzed using the chi-squared test for unpaired data and McNemar's test for paired data. ns, not significant; p < .05; p < .005; p < .001; %, percentage; BL, baseline colonoscopy; FU, follow-up colonoscopy; -, negative; +, positive.

**Table 1:** Characteristics of ulcerative colitis (UC) patients according to CMV status of biopsies at baseline (BL).

		CMV negative (*n* = 118)	CMV positive (*n* = 63)	*p*-value
**general characteristics**	age*, median (IQR), years	35 (25-47)	41 (29-55)	**.006**
	male sex, *n* (%)	70 (59)	38 (60)	.896
	duration of disease, median (IQR), months	40 (12-113)	30 (9-85)	.51
**disease extent** **(Montreal)**	E1, *n* (%)	8 (7)	2 (3)	.185
	E2, *n* (%)	53 (45)	39 (62)	
	E3, *n* (%)	57 (48)	22 (35)	
**UC therapy**	5-ASA (p.o.), *n* (%)	71 (60)	38 (60)	.985
	5-ASA (p.r.), *n* (%)	20 (17)	8 (13)	.445
	prednisolone (p.o.), *n* (%)	49 (42)	20 (32)	.136
	budesonide (p.r.), *n* (%)	14 (12)	3 (5)	.237
	azathioprine, *n* (%)	21 (18)	10 (16)	.468
	anti-TNF, *n* (%)	13 (11)	8 (13)	.168
	vedolizumab, *n* (%)	1 (1)	1 (2)	.658
	ustekinumab, *n* (%)	1 (1)	0 (0)	.354
	ozanimod, *n* (%)	0 (0)	0 (0)	
	jak-inhibitors, *n* (%)	0 (0)	1 (0)	.145
**No. of previous failures of advanced therapy**	0, *n* (%)	104 (88)	50 (79)	.123
	1, *n* (%)	11 (9)	11 (18)	
	2, *n* (%)	3 (3)	2 (3)	
	3, *n* (%)	0 (0)	0 (0)	
	4, *n* (%)	0 (0)	0 (0)	
	5, *n* (%)	0 (0)	0 (0)	
**eMayo Score**	0/1,* n* (%)	33 (28)	11 (18)	**.03**
	2, *n* (%)	44 (37)	21 (33)	
	3, *n* (%)	41 (35)	31 (49)	
**tMayo Score**	0, *n* (%)	18 (15)	5 (8)	**.011**
	1, *n* (%)	25 (21)	8 (13)	
	2, *n* (%)	65 (55)	39 (62)	
	3, *n* (%)	10 (9)	11 (18)	
**CMV copies/ml eluate**	low (<1.000), *n* (%)		25 (40)	
	medium (1.000-10.000),* n* (%)		11 (17)	
	high (>10.000), *n* (%)		27 (43)	
**CMV therapy**	total, *n* (%)		38 (60)	
	ganciclovir, IV, *n* (%)		1 (2)	
	valganciclovir, p.o.,* n* (%)		12 (19)	
	ganciclovir/valganciclovir, *n* (%)		25 (40)	
	duration, median (IQR), days		32 (28-56)	

CMV, cytomegalovirus; 5-ASA, 5-aminosalicylic acid; p.o., per os; p.r., per rectum; n, number; E1, proctitis; E2, left-sided colitis; E3, extensive colitis/pancolitis; anti-TNF, infliximab, adalimumab, golimumab; eMayo, endoscopic Mayo score; tMayo, total Mayo score. Categorical variables were compared using the chi-squared test, and continuous variables were compared using the Mann-Whitney U test. All statistical tests were two-sided, and a p value < 0.05 was considered statistically significant.

**Table 2:** Characteristics of ulcerative colitis (UC) patients according to CMV status of biopsies at the time of follow-up colonoscopy (FU).

		CMV negative (*n* = 118)	CMV positive (*n* = 63)	*p*-value
**Time from BL to FU, median (IQR), months**		45 (16-107)	34 (7-93)	.114
**UC therapy**	5-ASA (p.o.), *n* (%)	95 (81)	48 (76)	.5
	5-ASA (p.r.), *n* (%)	26 (22)	14 (22)	.977
	prednisolone (p.o.),* n* (%)	26 (22)	15 (24)	.495
	budesonide (p.r.), *n* (%)	16 (14)	14 (22)	.068
	azathioprine, *n* (%)	13 (20)	5 (8)	.581
	anti-TNF, *n* (%)	39 (33)	14 (22)	.255
	vedolizumab, *n* (%)	12 (10)	10 (16)	.1
	ustekinumab, *n* (%)	6 (5)	5 (8)	.452
	ozanimod, *n* (%)	0 (0)	1 (2)	
	jak-inhibitors,* n* (%)	1 (1)	4 (6)	.097
**No. of previous failures of advanced therapy**	0, *n* (%)	39 (33)	17 (27)	.302
	1, *n* (%)	40 (34)	20 (32)	
	2, *n* (%)	23 (20)	17 (27)	
	3, *n* (%)	11 (9)	5 (8)	
	4, *n* (%)	4 (3)	3 (5)	
	5, *n* (%)	1 (1)	1 (2)	
**eMayo Score**	0/1,* n* (%)	78 (66)	21 (33)	.086
	2, *n* (%)	26 (22)	18 (29)	
	3, *n* (%)	14 (12)	21 (38)	
**tMayo Score**	0, *n* (%)	66 (56)	20 (32)	**.001**
	1, *n* (%)	17 (14)	6 (10)	
	2, *n* (%)	28 (24)	33 (52)	
	3, *n* (%)	7 (6)	4 (6)	

CMV, cytomegalovirus; 5-ASA, 5-aminosalicylic acid; p.o., per os; p.r., per rectum; n, number; anti-TNF, infliximab, adalimumab, golimumab; eMayo, endoscopic Mayo score; tMayo, total Mayo score. Categorical variables were compared using the chi-squared test, and continuous variables were compared using the Mann-Whitney U test. All statistical tests were two-sided, and a *p* value < .05 was considered statistically significant.

## Abbreviations

CMV: cytomegalovirus
UC: ulcerative colitis
BL: baseline (colonoscopy)
FU: follow-up (colonoscopy)
PCR: polymerase chain reaction
RT-PCR: real-time polymerase chain reaction
DNA: deoxyribonucleic acid
IQR: interquartile range
5-ASA: 5-aminosalicylic acid
anti-TNF: anti-tumor necrosis factor (infliximab, adalimumab, golimumab)
JAK: Janus kinase
IBD: inflammatory bowel disease
tMayo: total Mayo score
eMayo: endoscopic Mayo score
eRemission: endoscopic remission
ceRemission: combined clinical and endoscopic remission
