# Supplementary material for: Cytomegalovirus Infection Is Associated with Increased Disease Activity and Worse Long-Term Course in Ulcerative Colitis
Source: Int J Med Sci. 2026 Sep 3;23(10):3262–70. doi: 10.7150/ijms.139439 (PMC13617507; doi:10.7150/ijms.139439)
Supplement: Supplementary file 1 [file ijmsv23p3262s1.pdf]

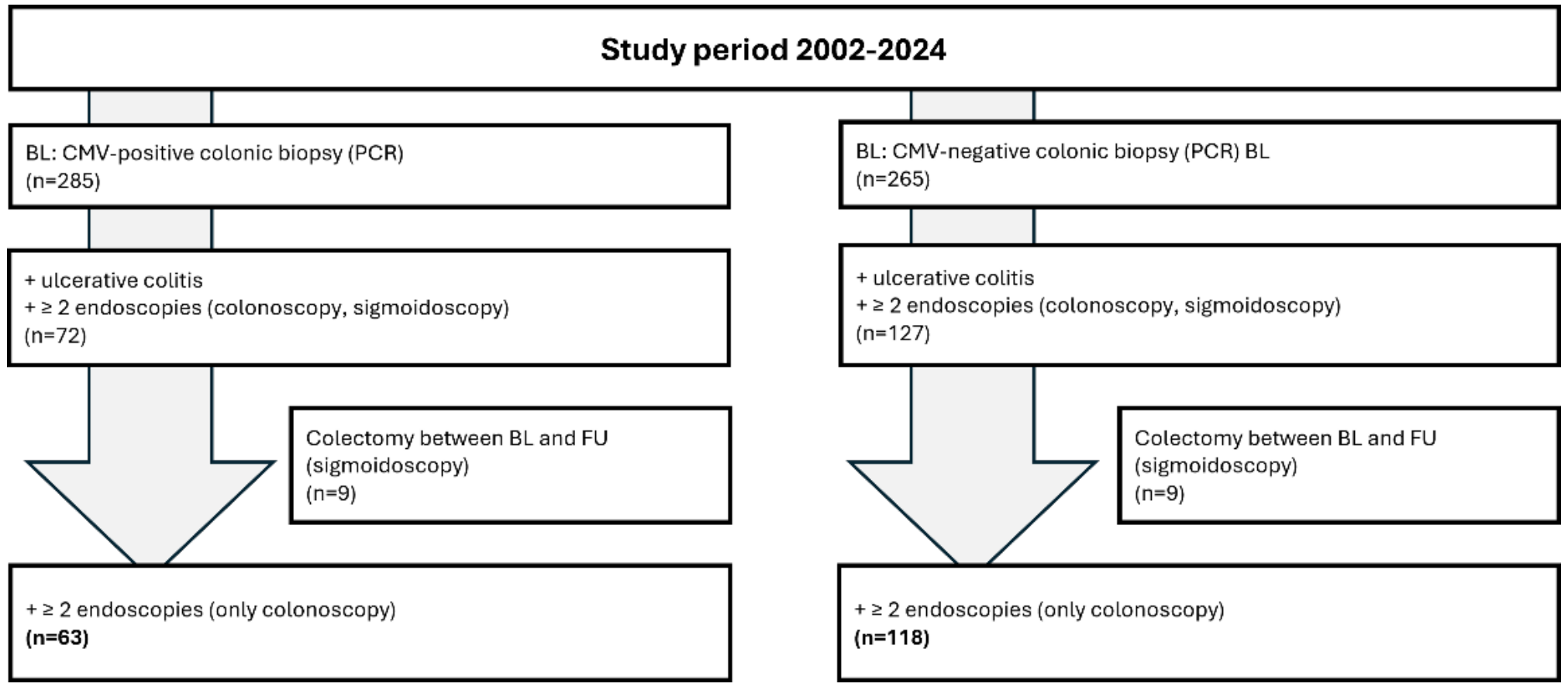

Supplemental Figure S1: Flowchart of the data extraction process. Baseline, BL; follow-up, FU; CMV, Cytomegalovirus; PCR, polymerase chain reaction; n, number.
